# Lipidomic Alterations and PPAR*α* Activation Induced by Resveratrol Lead to Reduction in Lesion Size in Endometriosis Models

**DOI:** 10.1155/2021/9979953

**Published:** 2021-09-11

**Authors:** Zhengyun Chen, Chunyan Wang, Cuicui Lin, Lifeng Zhang, Huimei Zheng, Yong Zhou, Xiaoyong Li, Chen Li, Xinmei Zhang, Xiaohang Yang, Minxin Guan, Yongmei Xi

**Affiliations:** ^1^The Women's Hospital, Zhejiang University School of Medicine, Hangzhou, Zhejiang 310006, China; ^2^Institute of Genetics, Zhejiang University, Hangzhou, Zhejiang 310058, China; ^3^Department of Human Genetics, Zhejiang University School of Medicine, Zhejiang Provincial Key Laboratory of Genetic & Developmental Disorders, Hangzhou, Zhejiang 310058, China; ^4^Joint Institute of Genetics and Genomic Medicine between Zhejiang University and University of Toronto, Zhejiang University, Hangzhou, Zhejiang 310058, China; ^5^Division of Medical Genetics and Genomics, the Children's Hospital, Zhejiang University School of Medicine, Hangzhou, Zhejiang 310006, China

## Abstract

Endometriosis is an estrogen-dependent chronic inflammatory disease that affects approximately 10% of women of reproductive age and up to 50% of women with infertility. The heterogeneity of the disease makes accurate diagnosis and treatment a clinical challenge. In this study, we generated two models of endometriosis: the first in rats and the second using human ectopic endometrial stromal cells (HEcESCs) derived from the lesion tissues of endometriosis patients. We then applied resveratrol to assess its therapeutic potential. Resveratrol intervention had significant efficacy to attenuate lesion size and to rectify aberrant lipid profiles of model rats. Lipidomic analysis revealed significant lipidomic alterations, including notable increases of sphingolipids and decreases of both glycerolipids and most phospholipids. Upon resveratrol application, both proliferation capacity and invasiveness parameters decreased, and the early apoptosis proportion increased for HEcESCs. The activation of PPAR*α* was also noted as a factor potentially contributing to recovery from endometriosis in both models. Our study provides valuable insight into the mechanisms of resveratrol in endometriosis and therefore strengthens the potential for optimizing resveratrol treatment for this disease.

## 1. Introduction

Endometriosis (EMs) is a refractory disease that affects approximately 10% of women of reproductive age and up to 50% of women with infertility. It is associated with functional endometrial glands and stroma implantation outside the uterus. Women with EMs often suffer from severe pelvic pain, resulting in significantly decreased quality of life and high costs for the healthcare system [1]. Currently available treatments include surgery and hormone medication, both of which have many unpleasant side effects and a high rate of relapse beyond their completion [2, 3]. Searching for new and long-term effective treatment for endometriosis is of great significance.

Resveratrol (trans-3,5,4′-trihydroxystilbene), a phytoalexin polyphenol found in natural plants or fruits, has previously been highlighted as a potential supplement for the treatment of cancers, cardiovascular disease, and EMs [4–7]. The pharmacological effects of resveratrol on the energy and lipid metabolism have been revealed in animal models or in human eutopic endometrial stromal cells (HESCs) of EMs [8]. Resveratrol intervention has also led to a decrease in total cholesterol and triacylglycerol concentrations in individuals with dyslipidemia [9, 10]. High levels of serum Lp(*α*), TG, and ApoA1, but not LDL-C nor HDL-C, have been identified in EMs patients [11–13]. However, such serum metabolite indices seemed to lack specificity and sensitivity for the diagnosis of EMs. Lipidomic analysis has been recently used to assess lipid homeostasis in various disease conditions [14]. In such studies, elevated levels of SM, PC, and TG were identified in serum samples of ovarian endometrioma patients [15]. Significant alterations in SM, PC, TG, and PE between the eutopic and ectopic endometriums were also noted in patients with EMs [16, 17]. As a direct infiltration environment for ectopic endometrium, peritoneal fluid in patients with EMs was found to have a decreased PC level [18, 19]. So far, such lipidomic analysis data has been predominantly targeted simply to find biomarkers for the clinical detection of this disease. However, these observations may indicate that abnormal lipid distribution may actually play a more significant role in the pathology of EMs. Despite this, little attention has been given towards the potential application of lipidomic factors for use as a more direct disease target or for their potential in analysis of drug efficacy or further elucidating disease mechanisms.

In the present study, to explore the mechanisms associated with the development of endometriosis and the effects of resveratrol on the treatment of this disease, we generated two models: the first in rats and the second using human ectopic endometrial stromal cells (HEcESCs). The results showed that resveratrol had significant efficacy to rectify aberrant lipid profiles and to reduce the lesion size in model rats and also leading to significant lipidomic alterations in HEcESCs associated with reduced invasiveness and proliferation. Our study provides some novel insights into the molecular mechanisms of endometriosis and reveals the significant potential of resveratrol for the treatment of this disease.

## 2. Materials and Methods

### 2.1. Establishment of a Rat Model of EMs

#### 2.1.1. Animals

Fifty female Sprague-Dawley rats aged 8-10 weeks, weighing 200-250 g, were placed in a clean-level environment in the Zhejiang University Laboratory Animal Center with 12 hours light/dark cycles and regular feeding. Animal experimental methods and purposes were all in line with ethical standards and international practices.

#### 2.1.2. Modeling

Prior to any surgery, the estrous cycle stages of female rats were examined using vaginal biopsy samples. Attrition cells, shown as irregular keratinocyte-like cells and gathered together on the slides, were considered to be an indicator of a mature estrous stage for efficient EMs modeling. Rats having a 4- to 5-day estrous cycle and two consecutive estrus cycles were then selected for surgery. The animals were anesthetized using 45 mg/kg by intraperitoneal injection of 3% pentobarbital (BIOCAM) sodium and operated under strict aseptic conditions at a room temperature of 28-30°C. Rat estrus epithelial tissue with a 0.8 × 0.8 cm^2^ endometrium was autotransplanted into the abdominal wall. Welfare nursing was provided after the operation. Ten rats were also selected for a placebo operation to serve as the sham group. The animals were fed regularly for 4 weeks.

#### 2.1.3. Examination

Laparotomies were performed 4 weeks after the surgery. The rats were euthanized, and the laparotomy was performed to measure the size of the implants. Modeled rats were recorded, and the lesion volume was calculated using the following formula: *V* = *a* × *b*^2^/2 (where *a* represents the broadest transverse diameter of the lesion and *b* represents the vertical diameter line), and *V* ≥ 2 mm^3^ was considered as a successful model.

### 2.2. Resveratrol Treatment in Rat EMs Model

Resveratrol was dissolved in 35% DMSO for intraperitoneal injection in rats, while the sham group and EMs group were injected with the same amount of solvent (0.9% NaCl+35% DMSO). Thirty rats with successful modelling were divided into three groups randomly: EMs group (*n* = 10), Res-med group (*n* = 10, resveratrol dose = 15 mg/kg/d), and Res-high group (*n* = 10, resveratrol dose = 45 mg/kg/d). The control group was also operated and treated with solvent (sham, *n* = 10). The rats of the four groups were administered continuously for 28 days. Lesions were then examined (as the above method). Lesion tissues and blood were sampled before and after resveratrol treatment for evaluation.

### 2.3. HE Staining and Immunohistochemical Staining

Lesion tissues were fixed in 10% formalin and dehydrated with a gradient of alcohol for paraffin slicing. The sections were processed according to a standard protocol for staining with hematoxylin and eosin (Solarbio). Images were taken under a light microscope (Nikon), and pathological features were analyzed. For immunofluorescence, lesion tissues were immersed in 4% paraformaldehyde in PBS for 24 hours and then embedded in O.C.T. Compound (Thermo Fisher Scientific) after infiltrating with 30% sucrose overnight. Transverse sections of the lesions (10 *μ*M thick) were mounted onto slides and blocked with 20% fetal calf serum in PBS for 1 hour. They were incubated with primary antibody caspase 8 (Proteintech) at 4°C overnight and then the secondary antibodies Alexa Fluor 488 goat anti-rabbit IgG (Proteintech). Images were taken by a light microscope (Nikon), and proportion of the total area with positive immunohistochemistry was analyzed with ImageJ.

### 2.4. Detection of Serums TC, TG, HDL, and LDL of Rat Models

Whole blood (500 *μ*L) was collected and centrifuged to detect TC, TG, HDL, and LDL and with analysis using a fully automatic biochemical analyzer (Toshiba FR120). The following detection kits (BeijingBJ•XinChuangYuan BIOTECH CO., LTD.) were used: total cholesterol measurement kit (CHOD-PAP method), low-density lipoprotein cholesterol measurement kit (direct method-protective reagent method), high-density lipoprotein cholesterol measurement kit (direct method-selective inhibition method), and triglyceride kit (GPO-PAP).

### 2.5. 1Culturing of HEcESCs

With permission of the patients, lesion tissues from the 8 patients whose intraoperative r-ASRM scores were all endometrial stage 3/4 were sampled under sterile conditions and kept in cold DMEM/F-12 (Gibco) with 1 : 100 penicillin-streptomycin liquid (Beyotime) for subsequent cell culture. The tissues were digested with 0.2% type I collagenase (Solarbio) in 37°C for 1.5 hours and then hand filtered using a 70 *μ*M cell filter (BD Falcon). Cells were cultured in DMEM/F-12 with 10% FBS (Gibco) and 1% penicillin-streptomycin liquid in an incubator (ESCO) at 37°C, 5% CO_2_. The cells were passaged when the density of primary cells had reached more than 75%. Resveratrol (Selleck) was initially dissolved in DMSO (Sangon) to make 20 mM and 8 mM mother fluids and then diluted to the working concentrations of 100 *μ*M and 40 *μ*M, respectively.

### 2.6. Lipidomic Analysis

#### 2.6.1. Sample Preparation and Detection

Eight groups of the cultured primary HEcESCs were divided into two parts. One was treated with 100 *μ*M resveratrol in medium, and the other was treated with solvent only. Culturing was for 48 hours where about 1 × 10^6^ cells were collected and subjected to the following lipidomic analysis. All samples were prepared according to the previously described techniques [20, 21]. An UHPLC system was used to coordinate an electrospray ion source using a Q Exactive HF MS system (Thermo) which was used for lipid profiling ultra performance liquid chromatography-tandem mass spectrometry (UPLC-MS). Chromatographic conditions are as follows: flow rate was 0.26 mL/min while column temperature was 55°C. The mobile phases consisted of (a) 60% acetonitrile/H_2_O with 10 mM ammonium and (b) isopropanol : acetonitrile = 9 : 1 (with 10 mM ammonium format). We applied positive and negative mode linear gradients to detect the subjects, respectively. Mass spectrometry was performed using a Thermo Q Exactive^™^ benchtop Orbitrap mass spectrometer equipped with heated ESI source in ESI-positive and ESI-negative modes (Thermo).

#### 2.6.2. Data Processing

All assay raw data were collected using Xcalibur data acquisition software (Thermo). The data, including *m*/*z*-values, retention times, and peak areas, were extracted using LipidSearch software (Thermo). All of the detected lipids were quantified using the Thermo TraceFinderEFS software (version 3.2). The lipid molecules were named by reference to the LIPID MAPS website. We enabled One Map (http://www.5omics.com/) software to support comprehensive metabolic data analysis. Multivariate statistical analysis was performed online. This included hierarchical clustering analysis, Pearson correlation heat maps, *Z*-score plot, volcano plot, principal component analysis (PCA), partial least squares discriminant analysis (PLS-DA), orthogonal partial least squares discriminant analysis (OPLS-DA), construction of a receiver operating characteristic curve (ROC) univariate, and the construction of a permutation plot and a variable importance in projection (VIP) plot.

### 2.7. Proliferation, Invasiveness, and Apoptosis Assays

#### 2.7.1. Cell Proliferation Assay

Cultured HEcESCs, grown to the logarithmic growth stage, were digested with 0.25% trypsin-EDTA and resuspended. 100 *μ*L of 4 × 10^4^ cells/mL suspension was inoculated into 96-well plates (Corning) for 24 hours. Four experimental groups, each with triplets, were prepared as follows: blank (100 *μ*L culture medium), control (DMSO), Re-40 *μ*M (resveratrol at a concentration of 40 *μ*M), and Re-100 *μ*M (resveratrol at a concentration of 100 *μ*M). After 48 hours of treatment, 10 *μ*L CCK8 (Solarbio) solution was added and incubated for another 4 hours. The cell viability was measured with a BioTek Synergy 1 plate reader (BioTek) and calculated.

#### 2.7.2. Cell Invasiveness Assay

The Matrigel (Solarbio) was thawed at 4°C and diluted with 1 : 12 in serum-free DMEM/F-12. The 8 *μ*M upper chamber of the Transwell plates (Corning) was coated and gelatinized for 1 hour in an incubator at 37°C. The cells were treated with DMSO, 40 *μ*M resveratrol, and 100 *μ*M resveratrol for 48 hours and then digested. The upper chambers were filled with 2 × 10^4^ cells in 1% FBS DMEM/F-12 medium and the lower chamber with 600 *μ*L 10% FBS DMEM/F-12 medium. The triple Transwell plates were placed at 37°C, in a 5% CO_2_ incubator for 48 hours. Transwell chambers were fixed with 95% ethanol and stained with 0.1% crystal violet for 30 minutes. Five visual fields (400 x) were randomly selected under the microscope to count the cells that had crossed the Matrigel.

#### 2.7.3. Cell Apoptosis Assay

Cells were treated separately with DMSO, 40 *μ*M resveratrol, 100 *μ*M resveratrol, 100 *μ*M fenofibrate, or 50 *μ*M fenofibrate+20 *μ*M resveratrol for 48 hours and then digested and resuspended using a binding buffer (Beyotime) to make a 1 × 10^6^ cells/mL suspension. 100 *μ*L cell suspension was added into a 5 mL flow tube, and 5 *μ*L annexin V Alexa Fluor 488 was then added. The mixture was incubated in a dark room for 5 minutes, and 10 *μ*L PI and 200 *μ*L PBS were then added. Cellular apoptosis was analyzed by NovoCyte flow cytometer (ACEA).

### 2.8. Fenofibrate Treatment

Fenofibrate was dissolved in DMSO, the rats were intraperitoneally injected, and the sham group and EMs group were injected with the same amount of solvent. Thirty rats successfully modeled were randomly divided into four groups: EMs group (*n* = 10), Res group (*n* = 10, resveratrol dose = 45 mg/kg/d), and PPAR*α* group (*n* = 10, fenofibrate dose of 15 mg/kg/d). The rats were given the drug for 28 consecutive days. Lesions were measured after treatment.

### 2.9. PPAR-*α* Overexpression Plasmid Construction and Cell Transfection

Plasmid GV146 (CMV-MCS-IRES-EGFP-SV40-neomycin) was purchased from Shanghai Genechem. The PPAR-*α* coding sequence (CDS) was amplified using primers listed below and cloned into a GV146 vector. The sequence of the successful clone was confirmed by DNA sequencing: PPAR*α* (18191-1)-P1: TACCGGACTCAGATCTCGAGCGCCACCATGGTGGACACGGAAAGCCC; PPARA(18191-1)-P2: TACCGTCGACTGCAGAATTCTCAGTACATGTCCCTGTAGATCTC. hEM15A was purchased from Shanghai Chunmai Biotechnology Co., Ltd. Cells were maintained in 1640 medium supplemented with 10% FBS and incubated in a humidified chamber (ESCO) with 5% CO_2_ at 37°C. The cells were passaged when the density of primary cells had reached more than 75%. Plasmids encoding the PPAR-*α* overexpression plasmid were transfected into the cell using a Hieff Trans transfection reagent according to the protocol provided (Yeason, Shanghai).

### 2.10. qRT-PCR

Total RNAs were extracted from tissues or cells using TRIzol (Sangon) and then reversely transcribed into cDNA using a reverse transcription kit (Vazyme). ChamQSYBRqPCR Master Mix (Vazyme) was applied for qRT-PCR using a real-time quantitative PCR machine HT faster 9600T (Biosystem). The following primers were used (Supplementary Table 1).

### 2.11. Western Blot

Total proteins were extracted using a standard protein lysis buffer, protease, and phosphatase inhibitor mixture (Roche Diagnostics). The protein concentration was determined using a BCA kit (Gene Ray). The standard curve was made according to the absorption value of standard liquid, and the concentration of protein was measured and calculated. Samples were subjected to SDS-PAGE and transferred to a polyvinylidene fluoride membrane. Membranes were immunoblotted with primary antibodies overnight at 4°C and then incubated with secondary antibodies conjugated with HRP. The following antibodies were used: anti-PPAR*α* (1 : 500, Proteintech), anti-Actin (1 : 1000, Goodhere Biotechnology Co., AB-M-M001), anti-MMP2 (Abclonal, 1 : 1000), anti-VEGFA (Abcam, 1 : 1000), anti-BCL2 (Proteintech, 1 : 1000), anti-ICAM-1 (Abclonal, 1 : 1000), anti-GAPDH (Proteintech, 1 : 1000), anti-Phospho-AKT (Ser473) (Proteintech, 1 : 500), and anti-AKT (Proteintech, 1 : 500). Detection of proteins was performed using the ChemiLucent™ ECL detection reagents (Millipore, WBKLS0500). Images were taken using the chemiluminescence imaging system (Clinx Science Instruments).

### 2.12. The Open Field Assay

The open field experiment was carried out in a market equipment (open square box, 2 m × 2 m × 50 cm) which was equipped with an infrared camera (CCTVLENS). Experiments were performed in the standard manner. The rats were placed in the test room to acclimatize for 2 hours, and then, each one was placed in the same orientation when entering into the market. The test time for each rat was 5 minutes. The trajectory and movements of the rats were tracked using VideoTrack 3.10 software for subsequent analysis of parameters such as the movement time in the central area and the number of entrances into the central area. In between each experiment, the field was cleaned with 70% ethanol to eliminate the odour of the previous rat.

### 2.13. Statistics

Data are expressed as the mean ± standard error of the mean (SEM). Statistical analyses were conducted using GraphPad Prism 5. Comparisons between groups were made by Student's *t*-test. Differences were considered significant at a *p* value of <0.05, marked as ^∗^*p* < 0.05, ^∗∗^*p* < 0.01, ^∗∗∗^*p* < 0.001, and ^∗∗∗∗^*p* < 0.0001.

## 3. Results

### 3.1. Establishment of a Rat Model of Endometriosis and the Therapeutic Effect of Resveratrol

**Figure 1:**
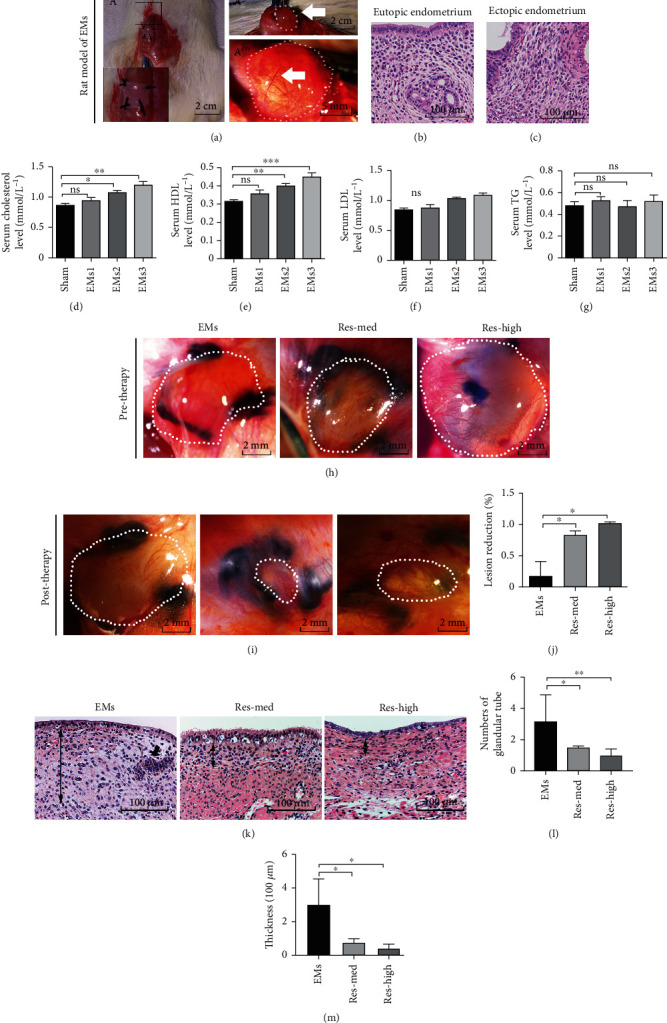
Resveratrol attenuated the lesions of endometriosis model rats. (a) Autotransplantation of rat endometrium by fixation of uterine tissue samples to the abdominal wall (A'). After 4 weeks of modelling, successful implants (white arrow) showed the macroscopic appearance of endometriotic implants (A”-A”'). Scale bar: 2 cm and 5 mm. (b, c) HE staining of implant-derived ectopic endometrium and eutopic endometrium. Scale bar: 100 *μ*M. (d–g) Serum levels of cholesterol (d), HDL (e), LDL (f), and TG (g) in animal modeling: experimental endometriosis model with different sizes of lesions: 2 mm^3^ ≤ EMs1 < 20 mm^3^, 20 mm^3^ ≤ EMs2 < 100 mm^3^, or EMs3 ≥ 100 mm^3^, each *n* ≥ 5. (h, i) Reduced size of endometriosis lesions after 4 weeks of resveratrol treatment. Res-med group (*n* = 10, resveratrol dose = 15 mg/kg/d), Res-high group (*n* = 10, resveratrol dose = 45 mg/kg/d), and EMs group (solvent control, *n* = 10). Scale bar: 2 mm. (j) Significant reduction of lesion sizes in both Res-med groups and Res-high groups is shown. (k–m) HE staining showing reduced numbers of glandular tube (black arrowheads in (g) and (h)) and epithelial thickness (black arrows in (g) and (i)) of the implanted endometrium in Res-med and Res-high groups. Scale bar: 100 *μ*M. Data are shown as the mean ± SEM. ^∗^*p* < 0.05 and ^∗∗^*p* < 0.01 by *t*-test. *N* = 10. EMs: endometriosis; Res: resveratrol.

To explore the mechanisms associated with the development of endometriosis, a rat model of endometriosis was established using autologous transplantation of rat estrus epithelial tissue into the abdominal wall (Figure 1(a), A'). Examined after 4 weeks of modelling, successful implants showed EMs-like lesions appearing as vesicular cysts (Figure 1(a), A”), filled with clear or turbid yellow-brown liquid and surrounded by connective tissue and angiogenesis (Figure 1(a), A”'). HE staining showed that the pathological features of implant-derived ectopic endometrium shared similarities to the eutopic endometrium (Figures 1(b) and 1(c)).

To further evaluate the pathological characteristics of the model rats, we examined the serum metabolites including cholesterol, HDL, LDL, and TG of the model rats and the sham group of animals. The EMs model animals were classified into three levels according to severity (*n* = 10 in each group): EMs 1: 2 mm^3^ ≤ lesion volume < 20 mm^3^, EMs 2: 20 mm^3^ ≤ lesion volume < 100 mm^3^, and EMs 3: lesion volume ≥ 100 mm^3^. Compared to the sham group, in the EMs 1 group, there were no significant differences in serum cholesterol (Figure 1(d)), HDL (Figure 1(e)), LDL (Figure 1(f)), and TG (Figure 1(g)). In both the EMs 2 and EMs 3 groups (≥ 20 mm^3^), the levels of serum cholesterol, HDL, and LDL, but not TG, were significantly increased (Figures 1(d)–1(g)). These data indicated a positive correlation between the serum levels of cholesterol, HDL and LDL, and lesion severity in the model rats.

We also attempted to evaluate the anxiety of rats with EMs that were likely associated with pain and discomfort. An animal open field assay, often used as test for anxiety and the animal behavioral responses, was applied which could be scored by measuring the time spent in the center zone and by numbering the occasions where the rats crossed the central region [22]. The results indicated that the EMs rats group showed more anxiety than the control group (Supplementary Figures 1A and 1B), with significant decreases in the time spent in the central area (Supplementary Figure 1C) and decreased frequency of entering the central area (Supplementary Figure 1D).

To analyze the effects of resveratrol on model rats, 4 weeks after the treatment administration, the ectopic endometrial lesions of the model rats were examined. Compared to the EMs group without resveratrol treatment (Figures 1(h) and 1(i)), significant reduction of lesion size was shown in both the Res-med groups (medium dose of resveratrol) and Res-high groups (high dose of resveratrol) (Figure 1(j)). The pathological lesions of EMs are typically characterized by the histological accumulation of endometrial epithelial, glandular tubes, and significant invasive growth [23, 24]. Histochemical staining (Figure 1(k)) showed that resveratrol treatment led to both significant decreases in glandular tubes (Figure 1(l)) and endometrial epithelial thickness (Figure 1(m)) in both the Res-med groups and Res-high groups, compared to the EMs group without resveratrol treatment.

### 3.2. Measurement of the Serum Lipid Profiles and Related Gene Expression of Model Rats upon Resveratrol Treatment

**Figure 2:**
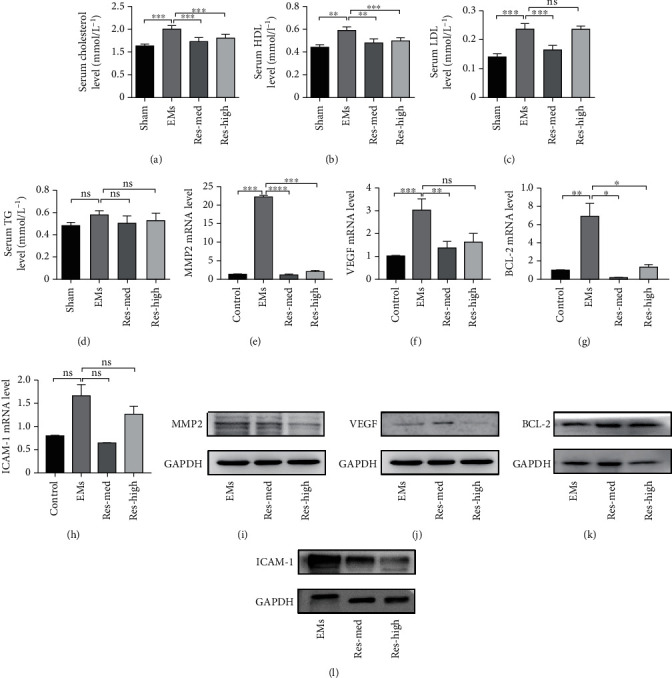
Changes in metabolite profiles and gene expression upon resveratrol treatment in rat models. (a–d) The levels of serum cholesterol (a), HDL (b), LDL (c), and TG (d) after resveratrol treatment. Each *n* = 10. (e–h) mRNA levels of MMP2 (e), VEGF (f), BCL-2(g), and ICAM1 (h) in ectopic endometrial lesions and upon resveratrol treatment were analyzed. (i-l) Changes of MMP2 (i), VEGFA (j), BCL-2 (k), and ICAM1 (l) protein levels after different concentrations of resveratrol treatment. GAPDH was used as a loading control. Data are shown as the mean ± SEM. ^∗^*p* < 0.05, ^∗∗^*p* < 0.01, ^∗∗∗^*p* < 0.001, and ^∗∗∗∗^*p* < 0.0001 by *t*-test. EMs: endometriosis; Res: resveratrol; HDL: high-density lipoprotein; LDL: low-density lipoprotein; TG: triglyceride; ns: no significance. Res-med: dose = 15 mg/kg/d; Res-high: dose = 45 mg/kg/d.

After resveratrol treatment for 4 weeks, we measured the serum cholesterol (Figure 2(a)), HDL (Figure 2(b)), LDL (Figure 2(c)), and TG (Figure 2(d)) of model rats. Results showed that the levels of cholesterol, HDL, and LDL in the Res-med group and the levels of cholesterol and HDL, but not LDL, in the Res-high group were significantly decreased, compared to the EMs group without such treatment (Figures 2(a)–2(c)). No significant changes in TG level were observed among these groups from that of controls (Figure 2(d)). These data suggested that resveratrol treatment has efficacy to rectify the aberrant lipid profiles in EMs model rats.

As the occurrence of EMs had been previously shown to be related to cell adhesion, angiogenesis, and apoptosis in a mouse model, as related to the expression of specific genes including MMP-2, ICAM-1, VEGF, and BCL-2 [9, 24, 25], we extracted mRNA and protein from lesion tissues of model rats to analyze any expression alterations associated with resveratrol. Results showed that the mRNA expressions of MMP-2, VEGF, and BCL-2, but not ICAM-1, were significantly increased in lesion tissues of the EMs group as compared to the sham group. After resveratrol treatment, the mRNA expressions of MMP-2, VEGF, and BCL-2, but not ICAM-1, were significantly decreased, compared to the EMs group (Figures 2(e) and 2(f)). We examined the protein expression levels of MMP-2, VEGF, BCL-2, and ICAM-1 of the lesion samples before and after resveratrol treatment. The protein expressions of MMP2 and ICAM-1 were significantly decreased after the treatment, which corresponded to their mRNA levels. However, the protein expressions of VEGF and BCL-2 showed various alterations after resveratrol treatment (Figures 2(i)–2(l)). These observations indicated that, in addition to the reduction of lesion size upon resveratrol treatment in the model rats, there were also associated decreases in cell invasion and adhesion.

### 3.3. Culture of HEcESCs and the Effects of Resveratrol on Cell Proliferation, Invasiveness, and Apoptosis

**Figure 3:**
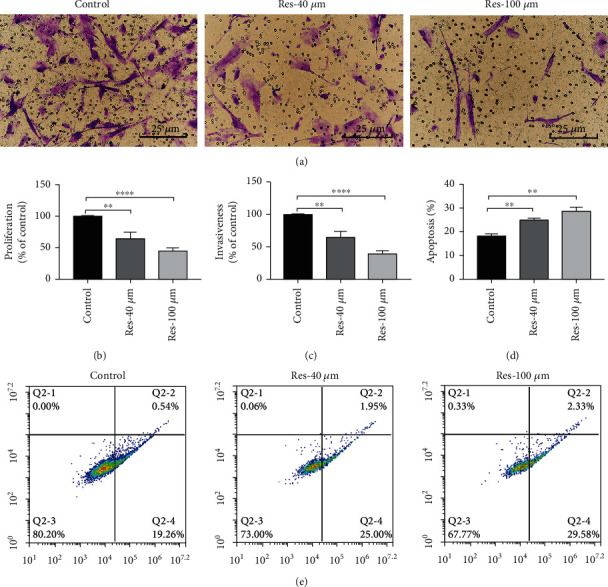
The effects of resveratrol on cell proliferation, invasiveness, and apoptosis in HEcESCs. (a) Analysis of the indicated invasion cells with Matrigel. (b) Statistical analysis of cell migration. (c) Decreased proliferation capacity of the HEcESCs after resveratrol treatment. (d) Statistical graph showing effect of resveratrol on apoptosis of HEcESCs. (e) Resveratrol increases HEcESCs apoptosis assessed by flow cytometric annexin-PI analysis. Resveratrol treatment at a concentration of 40 *μ*M or 100 *μ*M for 48 hours. Experiments in (a–e) were repeated at least 3 times, with similar results. Data are shown as the mean ± SEM.^∗∗^*p* < 0.01 and ^∗∗∗∗^*p* < 0.0001 by *t*-test.

To test the effects of resveratrol on lesion tissue of patients with EMs, we obtained lesion samples with permission from 8 patients undergoing laparoscopy surgery. The obtained HEcESCs were cultured and treated with or without resveratrol and then subjected to the assays for evaluation of cell proliferation, invasiveness (shown in Figure 3(a)), and apoptosis. After 48 hours of treatment with resveratrol at different concentrations (40 *μ*M and 100 *μ*M), the proliferation capacity of the HESCs had decreased by 36.30% in the Res-40 *μ*M group and 57.78% in the Res-100 *μ*M group, compared with the control groups: *p* < 0.01 (40 *μ*M) and *p* < 0.0001 (100 *μ*M) (Figure 3(b)). In the invasiveness assay, from the same amount of cells (2 × 10^4^), the number of cells then crossing the Matrigel was measured and differed significantly between the resveratrol treatment and control groups. At a concentration of 40 *μ*M or 100 *μ*M, a decrease of 35.00% (*p* < 0.01) and 61.72% (*p* < 0.001) was observed, respectively, compared with the control group (Figure 3(c)). The effects of resveratrol on the apoptosis of HESCs are shown in Figures 3(d) and 3(e). The proportion of early apoptosis in the control group was 19.26%, which increased to 25.00% after treatment with 40 *μ*M resveratrol and to 29.58% after treatment with 100 *μ*M resveratrol for 48 hours, both showing significant differences (*p* < 0.01) (Figures 3(d) and 3(e)).

### 3.4. Resveratrol Induced Lipidomic Alterations in HEcESCs

**Figure 4:**
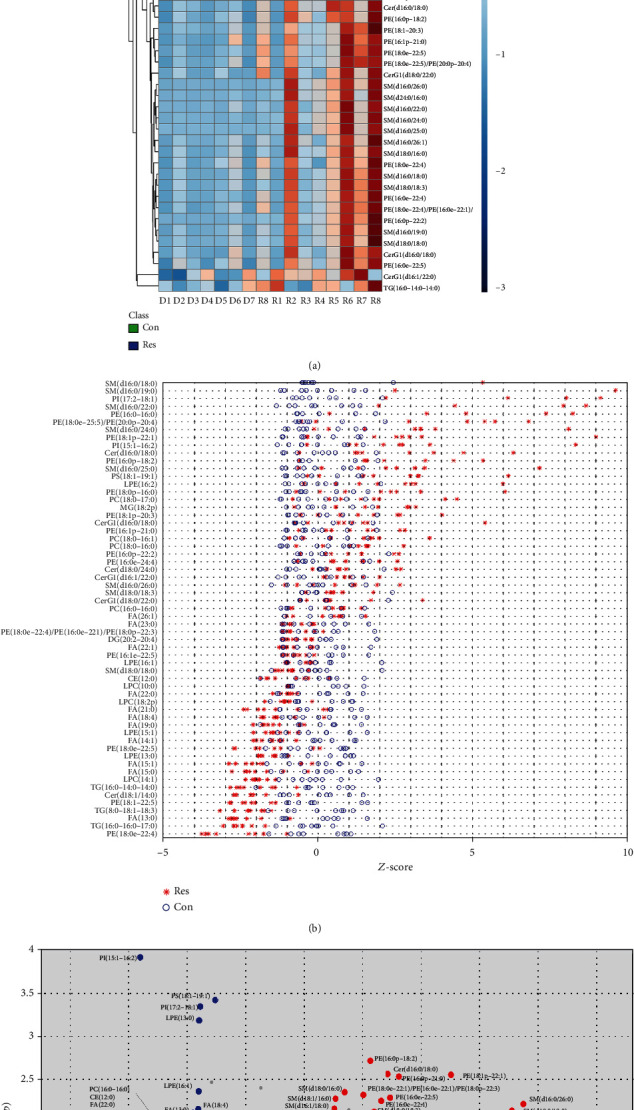
The effect of resveratrol treatment on lipid profiles in HEcESCs. (a) Heatmap representation of analytes in HEcESCs (*n* = 8) and HEcESCs treated with resveratrol (*n* = 8). Color scale indicates the relative richness of lipid metabolites. (b) *Z*-score quantification of lipids detected in both HEcESCs (Con) and HEcESCs treated with resveratrol (Res). A positive *Z*-score suggests possible upregulation, while a negative *Z*-score suggests possible reduction. (c) Volcano plot showing increased analytes (red) or decreased analytes (blue) in HEcESCs after resveratrol treatment versus HEcESCs without resveratrol treatment.

Resveratrol treatment in mouse models has been seen to protect against metabolic disease by activating SIRT1 and PGC-1 [26]. Resveratrol has also been seen as activating Sirt1 and PPAR*α* in rat endothelial cells which were able to sense fatty acids [27–29]. Thus, we considered the exploration of lipidomic changes in HEcESCs upon resveratrol treatment. Eight groups of primary HEcESCs treated with resveratrol for 48 hours (Res groups), together with those treated with DMSO as controls (Con groups), were subjected to lipid extraction and nontarget lipidomic analysis by UPLC-MS. Based on the OSI/SMMS lipid library, 809 qualitative lipid structures were differentially classified, mainly including 5 types of glycerophospholipids (PC, PE, PG, PS, and PI), 4 types of sphingolipids (SM, Cer, HexCer, and Hex2Cer), 3 types of glycerolipids (MG, DG, and TG), and FA. 638 lipids under the positive ions model and 313 lipids under the negative ions model were recognized. Among these, 132 lipids were identified in both ion models. Compared to the peak values of differential lipids between the Res groups and Con groups, 63 lipids were quantified as significantly altered candidates upon resveratrol treatment (*p* < 0.05, FC > 1.5, and VIP > 1). Using One Map (http://www.5omics.com/), univariate data analysis showed the overall metabolite features with different variations among samples of the paired groups (Res vs. Con). PE (16 : 0p-18 : 2), SM (18 : 0/18 : 0), PC (18 : 0-18 : 1), FA (13 : 0/14 : 1/15 : 0/15 : 1), PI (17 : 2/18 : 1), and Cer (d18 : 1/14 : 0) were significantly altered in the Res group (Figure 4(a)).

The lipid changes between the paired groups are also shown with a *Z*-scores plot (Figure 4(b)). In particular, the sphingolipids (such as Cer and SM) showed obvious increase, and glycerolipids (such as DG and TG), FA, and most of the phospholipids including PC, LPC, PE, LPE, PG, PI, and PS showed significant reduction (Figure 4(c)). Multivariate statistical analysis revealed an obvious separate trend between the Res group and Con group (Supplementary Figures 2A–2E). Among all the lipids altered after resveratrol treatment, PI (15 : 1-16 : 2) showed the most variation contributing to the separation between the two groups, with the highest VIP (VIP = 2.88) (Supplementary Figure 2F).

### 3.5. Lipid-Associated Signaling Pathways Affected by Resveratrol Treatment

**Figure 5:**
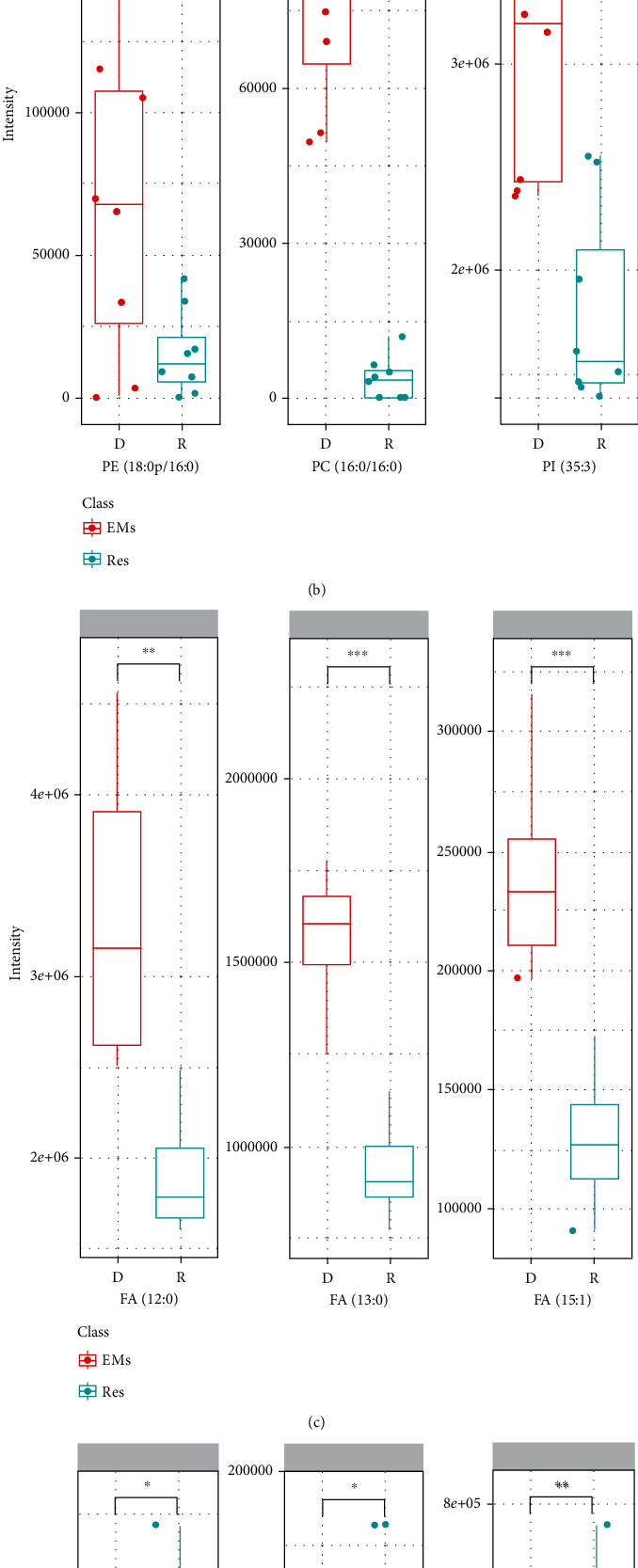
Lipid-associated signaling pathways affected by resveratrol treatment. (a) The altered lipid metabolites (under either VIP > 1, *p* < 0.05, or FC > 1.5 criteria) were subjected to the KEGG database for pathway enrichment analysis. The block represents the *p* value of the indicated pathways. (b–d) Key lipids FA (b), PE, PC, and PI (c), and Cer and SM (d) in the related signaling pathways that had significantly altered upon resveratrol treatment. Red: EMs group. Green: Res group.

The above altered lipid metabolites (under criteria either VIP > 1, *p* < 0.05, or FC > 1.5) were then subjected to the KEGG database for pathway enrichment analysis. As shown in Figure 5(a), among all related pathways, the lipidomic alterations upon resveratrol treatment were mostly assigned to the glycerophospholipid metabolism, insulin signaling, and sphingolipid signaling pathways, among all related pathways. Resveratrol could inhibit the synthesis of cholesterol and the downregulation of apolipoproteins [10]. The phospholipids PE, PC, and PI were significantly reduced upon resveratrol treatment (Figure 5(b)), which might result in a decreased synthesis of PI and PG in glycerophospholipid metabolism pathways and induced insulin signaling. Significant reduction of FA (Figure 5(c)) also affects cholesterol metabolism and induces insulin resistance. Significant increase of Cer and SM (Figure 5(d)) may be involved in the sphingolipid metabolism pathway. To characterize the molecular alterations in these specific metabolic pathways upon resveratrol treatment, we analyzed the protein levels of phosphorylated AKT, a downstream factor of insulin signaling [30], and mRNA levels of the key enzymes such as the PCYT1, CHPT1, CEPT1, and EPT1 of glycerophospholipid metabolism [31], the SPT, SGMS1, SMPD2, SPHK1, CERS2, DEGS1, and ACER1 of cholesterol metabolism [32], and the HMGCR, HMGCS1, ACAT2, NPC1L1, CYP7A1, and LDLR of sphingolipid metabolism [33]. Results showed that high dose of resveratrol significantly reduced the phosphorylation of AKT (Supplementary Figures 3A–3C). Significant alterations of mRNAs of the key enzymes corresponded with the lipidomic alterations assigned in the pathways of glycerophospholipid metabolism, cholesterol metabolism, or sphingolipid metabolism of HEcESCs upon resveratrol treatment (Supplementary Figures 3D–3F).

### 3.6. Resveratrol Induces PPAR*α* Activation in Both HEcESCs and Model Rats

**Figure 6:**
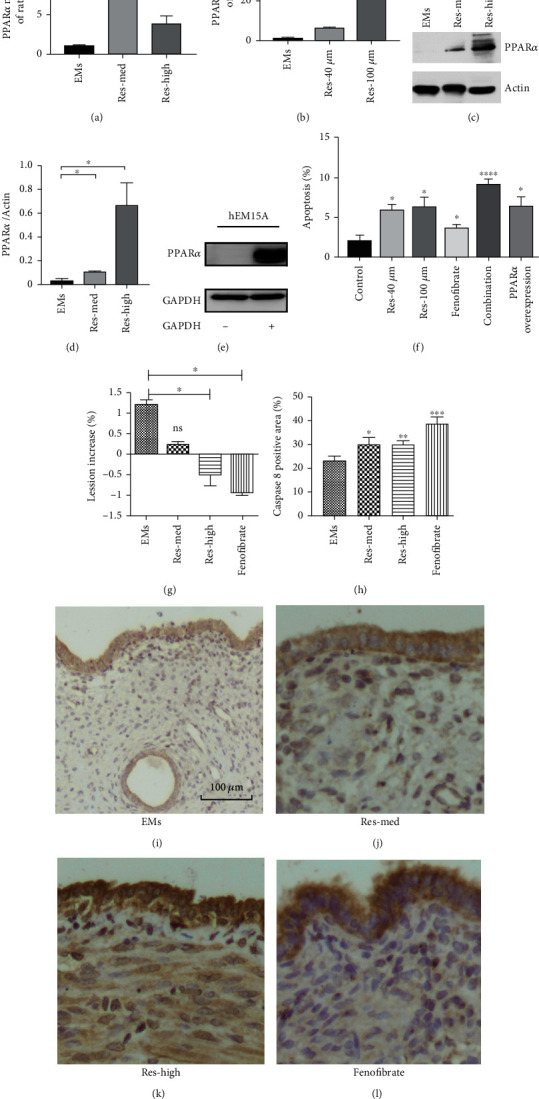
Resveratrol induces PPAR*α* activation in both HEcESCs and model rats. (a, b) Changes of PPAR*α* mRNA expression in the endometriotic implants of rat (a) and in the HEcESCs (b) after different concentrations of resveratrol treatment. (c, d) Detected changes of PPAR*α* protein levels in the endometriotic implants of rat detected. Actin was used as a loading control. (e) Western blot showed increased PPAR*α* protein levels after successful overexpression of PPAR*α* in hEM15A cells. GAPDH was used as a loading control. (f) Increased apoptosis capacity in hEM15A cells in the presence of 40 *μ*M or 100 *μ*M of resveratrol treatment, 100 *μ*M fenofibrates, a combination of resveratrol and fenofibrates treatment (50 *μ*M fenofibrate+20 *μ*M resveratrol), or PPAR*α*-overexpression. (g) Reduced size of endometriotic lesion after different concentrations of resveratrol and fenofibrate treatment for 28 days in rat models. (h–l) Immunohistochemical evaluation of caspase 8 expression of endometriotic implants in rat models. Increased expression of caspase8 endometriotic implants in rat models after resveratrol and fenofibrate treatment for 28 days. Scale bar: 100 *μ*M. Experiments were repeated at least 3 times, with similar results. Data are shown as the mean ± SEM. ^∗^*p* < 0.05, ^∗∗^*p* < 0.01, ^∗∗∗^*p* < 0.001, and ^∗∗∗∗^*p* < 0.0001 by *t*-test.

Resveratrol has been previously shown to stimulate PPAR*α* activation that suppresses the transcriptional activity of metabolic genes involved in energy and lipid metabolism homeostasis in endothelial cells [26, 27]. We analyzed the mRNA levels of PPAR*α* in HEcESCs and in ectopic endometrial tissues of the model rats upon resveratrol treatment. The mRNA expression of PPAR*α* was significantly increased in both model rats (Figure 6(a)) and HEcESCs (Figure 6(b)). The protein levels of PPAR*α* were analyzed using the ectopic endometrial tissues of model rats (EMs), and the lesion samples obtained from the model rats were treated with either medium or high dosage of resveratrol. An increased PPAR*α* expression was detected in the lesion tissues of model rats treated with resveratrol, compared to the untreated EMs groups (Figures 6(c) and 6(d)). We further constructed the recombinant plasmids encoding overexpression of PPAR*α* and transfected these into hEM15A cells (Figure 6(e)). We compared apoptosis of the hEM15A cells with PPAR*α* overexpression and of the groups treated with med-/high-resveratrol, the PPAR*α* agonist fenofibrate [28, 29], and the combination of fenofibrate and resveratrol in hEM15A cells. All showed to be able to induce a higher apoptosis in hEM15A cells (Figure 6(f)). This suggests that the effect of resveratrol on cell apoptosis is through the activation of PPAR*α*. We then used fenofibrate to treat rat models that might mimic the action of resveratrol. Surprisingly, the lesion sizes were significantly reduced after the treatment of the PPAR*α* agonist (Figure 6(g)). We have also examined the expression of caspase-8 by immunohistochemistry staining in lesions of rat models before and after the treatments, which showed a high expression of caspase upon either treatment of resveratrol or fenofibrate (Figures 6(h)–6(l)), indicating that apoptosis occurred. These observations indicate that lesion attenuation in model rats resulted by resveratrol might also occur via PPAR*α* activation.

## 4. Discussion

Endometriosis is a common gynecological condition characterized by endometrial glands and stroma at ectopic sites. Women with endometriosis often suffer from severe pelvic pain, resulting in significantly decreased quality of life and high costs for the healthcare system [1, 34]. Immune deficiency, heightened oxidative stress, and systemic chronic inflammation have been considered as critical facilitators in the progression of this disease [35, 36]. In the present study, we highlight the critical role of lipid metabolism in endometriosis. This may help identify patients at risk of developing this disease and aid in treatment decisions based on lipid profiles.

Lipids can function in tissue remodeling and act to maintain homeostasis during inflammatory processes [14]. Lp(*α*) acts as an acute-phase protein with a proinflammation role and is active in the modulation of tissue repair in cases of injury [37]. In the EMs model rats, serum levels of cholesterol, HDL, and LDL showed significant increases in a lesion size-dependent manner and subsequent significant decreases upon resveratrol treatment. These data manifested the critical involvement of lipid metabolites in EMs and the therapeutic efficacy of resveratrol targeting of the lipid metabolism.

As a natural supplement, resveratrol is believed to have multiple targets including cell membranes, intracellular receptors, signaling molecules, and various enzymes and transcription factors [20, 38–40]. Bruner-Tran et al., in their use of a nude mouse model, reported that resveratrol could inhibit the development of endometriosis and reduce the invasiveness of eutopic endometrial stromal cells [34]. Our own results also show that the treatment of resveratrol significantly inhibited proliferation, invasiveness, and increased apoptosis in HEcESCs, and this finding coincides with other studies on eutopic/ectopic endometrial stromal cells [41–43]. We also demonstrated that upon resveratrol treatment, glycerolipids such as FA, DG, and TG and most phospholipids showed significant reduction (Supplementary Figure 4), and this was particularly evident for those involved in the cholesterol metabolism and insulin signaling. Sphingolipids such as SM and Cer have been demonstrated to have an inhibitory effect on colon cancer, suppressing cell proliferation [44, 45]. In our experiments with resveratrol treatment of HEcESCs, increased SM and Cer were also observed along with the inhibition of cell proliferation (Supplementary Figure 4). These lipids are key components of the plasma membrane and other cellular compartments that integrate into many biological processes such as signaling pathways, wound healing, and anti-inflammation. Resveratrol-mediated lipidomic alterations may interplay with this dynamic network contributing to the attenuation of pathologies associated with endometriosis through changes in inflammatory response.

PPAR*α* responds to fatty acid signals derived from dietary lipids, pathogenic lipoproteins, or essential fatty acid metabolites and thereby controls both the lipid metabolism and inflammation [46–48]. In our experiment, we observed an increased expression of PPAR*α* in both rat endometriosis lesion and HEcESCs upon the treatment of resveratrol. Correspondingly, after treatment of resveratrol in HEcESCs, reduced FA might stimulate AMPK which could result in PPAR*α* activation and influence the regulation of lipid transport genes, such as ApoA1 and ApoA2 (Figure 5(b) and Supplementary Figure 4), being the main components of HDL to response inflammation [49]. Resveratrol-stimulated PPAR*α* activation has already been reported to be associated with an increased phosphorylation of AMPK in human glomerular endothelial cells [40]. In our study, lipidomic analysis of HEcESCs treated with resveratrol also showed a significant activation of PPAR*α*, probably also through an upregulated AMPK signaling and PCG1 pathway. Resveratrol-mediated reduction of DAG might directly induce the IRS/PI3K-AKT pathways (Supplementary Figure 4) [50]. Our study indicates that resveratrol functions as an agonist to stimulate PPAR*α* activation and may also interplay more directly with the lipid-associated pathways that contribute to the improvement in the prognosis of EMs (Supplementary Figure 4).

In conclusion, this comprehensive study showed that, in a rat model of endometriosis, the levels of serum metabolites such as CHOL, HDL, and LDL were positively correlated with the lesion severity. Resveratrol has a significant efficacy to rectify the aberrant lipid profiles and attenuate lesion size. Our study reveals that resveratrol may act through the lipid-associated mechanisms in which PPAR*α* could be a molecular target for the treatment of EMs.

## Data Availability

The datasets used or analyzed during the current study are available from the corresponding authors on reasonable request.

## Abbreviations

HMGCR: 3-Hydroxy-3-methylglutaryl-CoA
HMGCS1: 3-Hydroxy-3-methylglutaryl-CoA synthase 1
ACAT2: Acetyl-CoA acetyltransferase 2
ACER1: Alkaline ceramidase 1
Apo A1: Apolipoprotein A1
Cer: Ceramide
CERS2: Ceramide synthase 2
CEPT1: Choline/ethanolamine phosphotransferase 1
CYP7A1: Cytochrome P450 family 7 subfamily A member 1
DEGS1: Delta 4-desaturase, sphingolipid 1
CHPT1: Choline phosphotransferase 1
DG: Diacylglycerol
EMs: Endometriosis
HEcESCs: Ectopic endometrial stromal cells
EPT1: Ethanolaminephosphotransferase 1 homolog
FA: Fatty acids
FC: Fold change
HDL: High-density lipoprotein
HDL-C: High-density lipoprotein cholesterol
LDL: Low-density lipoprotein
Lp(*α*): Lipoprotein *α*
LDLR: Low-density lipoprotein receptor
LPE: Lysophosphatidylthanolamine
LDL-C: Low-density lipoprotein cholesterol
LPC: Lysophosphatidylcholine
MG: Monoacylglycerol
NPC1L1: NPC1-like intracellular cholesterol transporter 1
O-PLS-DA: Orthogonal partial least squares discriminant analysis
PCA: Principal component analysis
PLS-DA: Partial least squares discriminant analysis
PCYT1: Phosphate cytidylyltransferase 1A
PI: Phosphatidylinositol
PG: Phosphatidyl glycerol
PS: Phosphatidylserine
PC: Phosphatidylcholine
PE: Phosphatidylethanolamine
ROC: Receiver operating characteristic curve
Res: Resveratrol
SM: Sphingomyelin
SPT: Salivary protein cluster
SGMS1: Sphingomyelin synthase
SMPD2: Sphingomyelin phosphodiesterase 2
SPHK1: Sphingosine kinase 1
TG: Triglyceride
TC: Total cholesterol
VIP: Variable importance in projection.

## Supplementary Materials

Supplementary Materials Supplementary Figure 1: behavioral evaluation of model rats. Supplementary Figure 2: results of multivariate statistical analysis. Supplementary Figure 3: molecular characterization of related pathways. Supplementary Figure 4: molecular network mediated by resveratrol. Supplementary Table 1: a list of primers.
