# Supplementary material for: Lipidomic Alterations and PPARα Activation Induced by Resveratrol Lead to Reduction in Lesion Size in Endometriosis Models
Source: Oxid Med Cell Longev. 2021 Sep 11;2021:9979953. doi: 10.1155/2021/9979953 (PMC8452402; doi:10.1155/2021/9979953)
Supplement: Supplementary Materials — Supplementary Figure 1: behavioral evaluation of model rats. Supplementary Figure 2: results of multivariate statistical analysis. Supplementary Figure 3: molecular characterization of related pathways. Supplementary Figure 4: molecular network mediated by resveratrol. Supplementary Table 1: a list of primers. [file 9979953.f1.zip › 9979953.f1/9979953.f1.docx]

**Supplementary Figures and Figure legends**

**
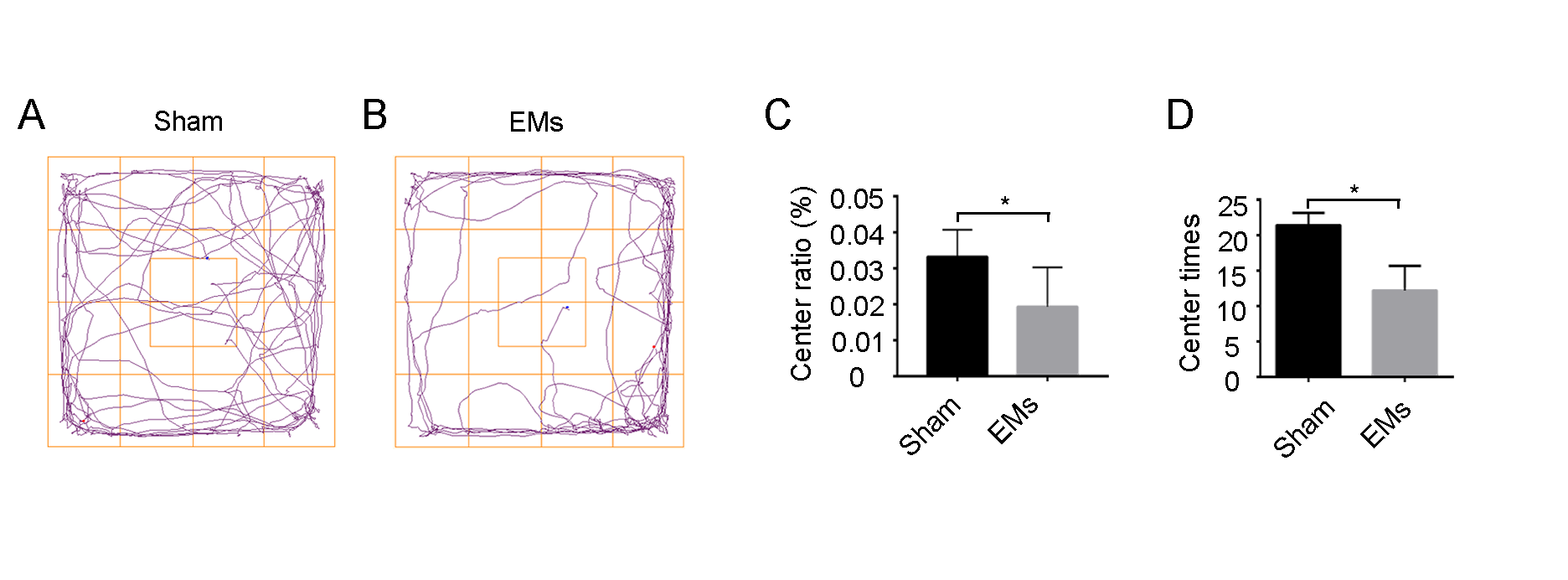
**

**Supplementary Figure 1.** **Behavioral evaluation of model rats. (A-B)** The open field experiment was used as a behavioral test to measure the movement and anxiety of animals under a certain condition. The rats with endometriosis or control animals (n ≥ 10) were placed in standard experimental conditions. **(C-D)** The movement distance (C) and frequency of entering the central area were recorded and analyzed within a 300 second period (D).


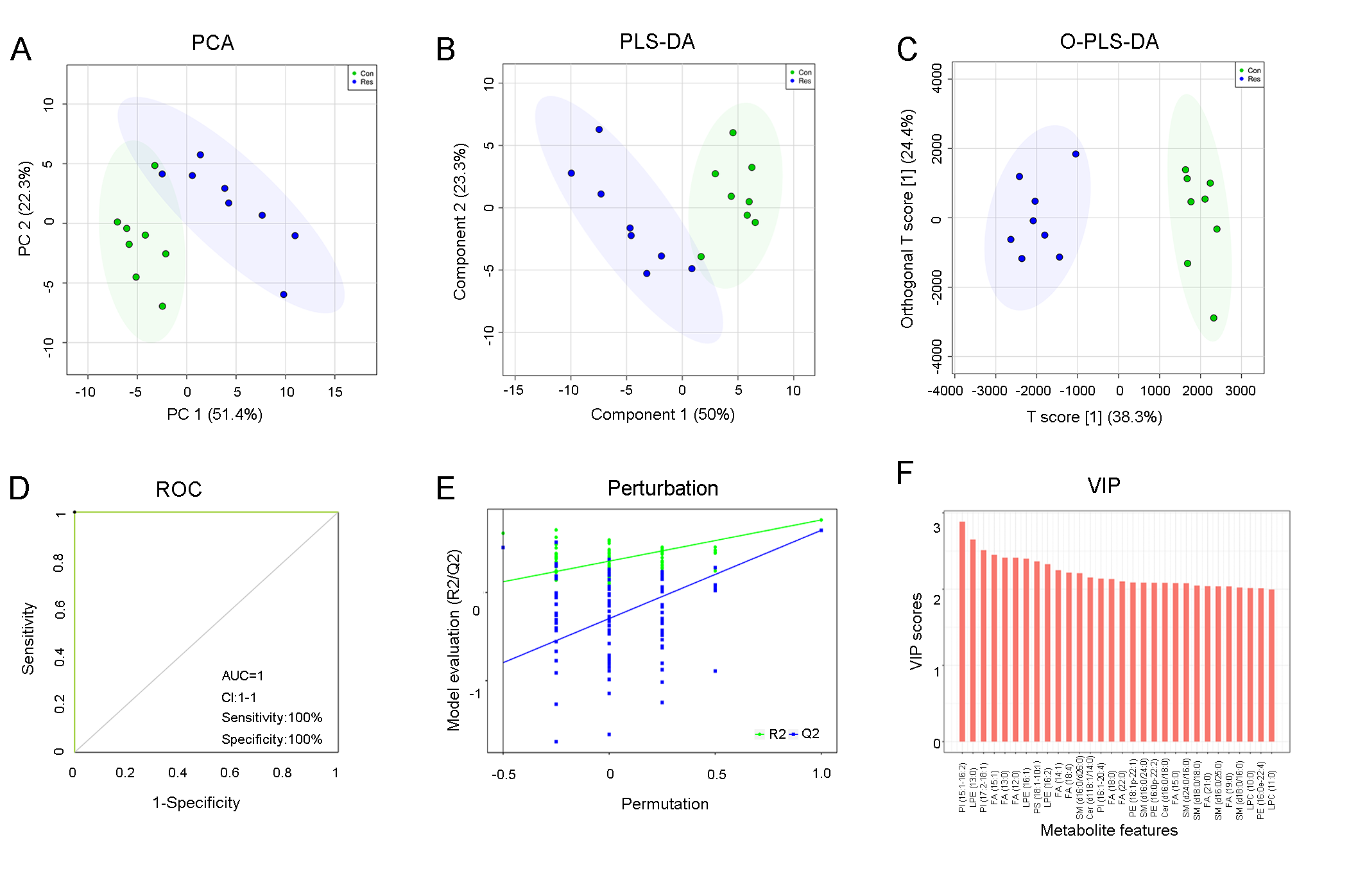


**Supplementary Figure 2.** **Results of multivariate statistical analysis. (A)** Principal component analysis (PCA) and clustering for HEcESCs samples from Con and Res. PCA score plot across the first 2 components created using log-transformed feature intensities across all metabolite features. **(B)** Partial least squares discriminant analysis (PLS-DA) of HEcESCs lipid profiles, 2D score plot. **(C)** Orthogonal projections to latent structures discriminant analysis (OPLS-DA) of HEcESCs samples (Con and Res). **(D)** ROC analysis was used to examine the property of the OPLS-DA model with AUC=1. **(E)** The permutation plot showing the best predictability and reliability of OPLS-DA model. **(F)** VIP plot. Metabolites were ranked according to their increasing importance to group separation between Control (Con) and Resveratrol treatment (Res).


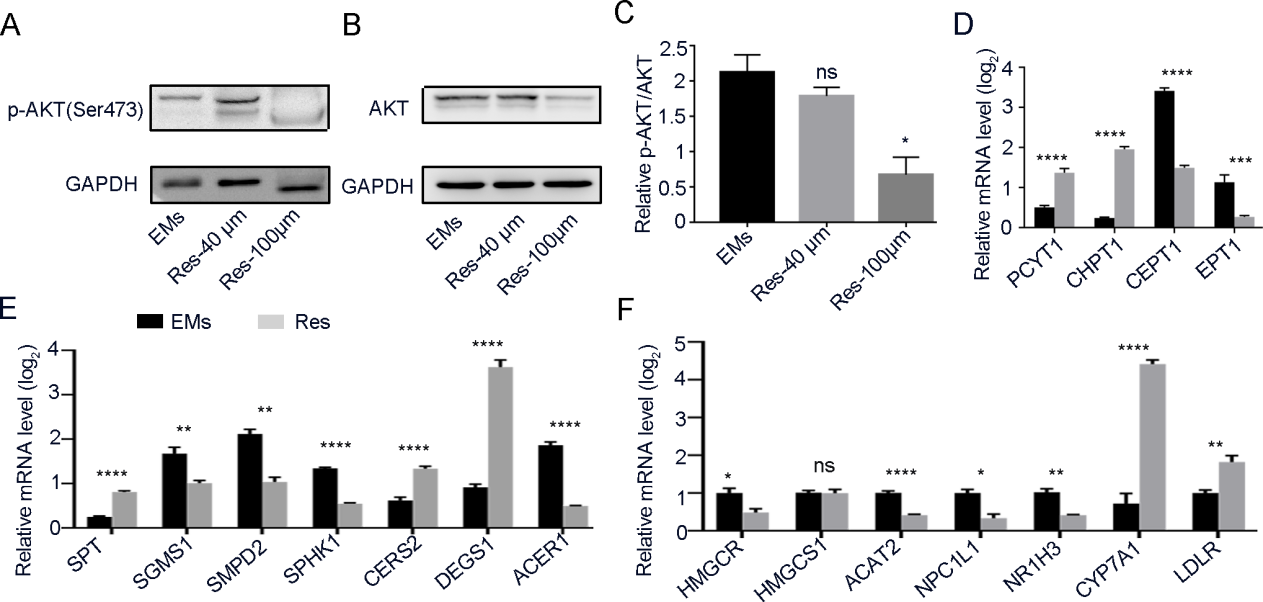


**Supplementary Figure 3.** **Molecular characterization of related pathways. (A-B)** Western blot showed p-AKT (S473) and AKT protein levels in ectopic endometrial lesions and upon resveratrol treatment. **(C)**. Statistical graph of measurement of relative protein level of pAKT/AKT. **(D)** Glycerophospholipid metabolic related enzymes: mRNA levels of PCYT1, CHPT1, CEPT1 and EPT1 in ectopic endometrial lesions and upon resveratrol treatment were analyzed. **(E)** Sphingolipid metabolic related enzymes: mRNA levels of SPT, SGMS1, SMPD2, SPHK1, CERS2, DEGS1 and ACER1 in ectopic endometrial lesions and upon resveratrol treatment were analyzed. **(F)** Cholesterol metabolic related enzymes: mRNA levels of HMGCR, HMGCS1, ACAT2, NPC1L1, CYP7A1 and LDLR in ectopic endometrial lesions and upon resveratrol treatment were analyzed. each n ≥3. Data are shown as mean ± SEM, ∗p < 0:05, ∗∗p < 0:01, ∗∗∗∗p < 0:0001 by T test.


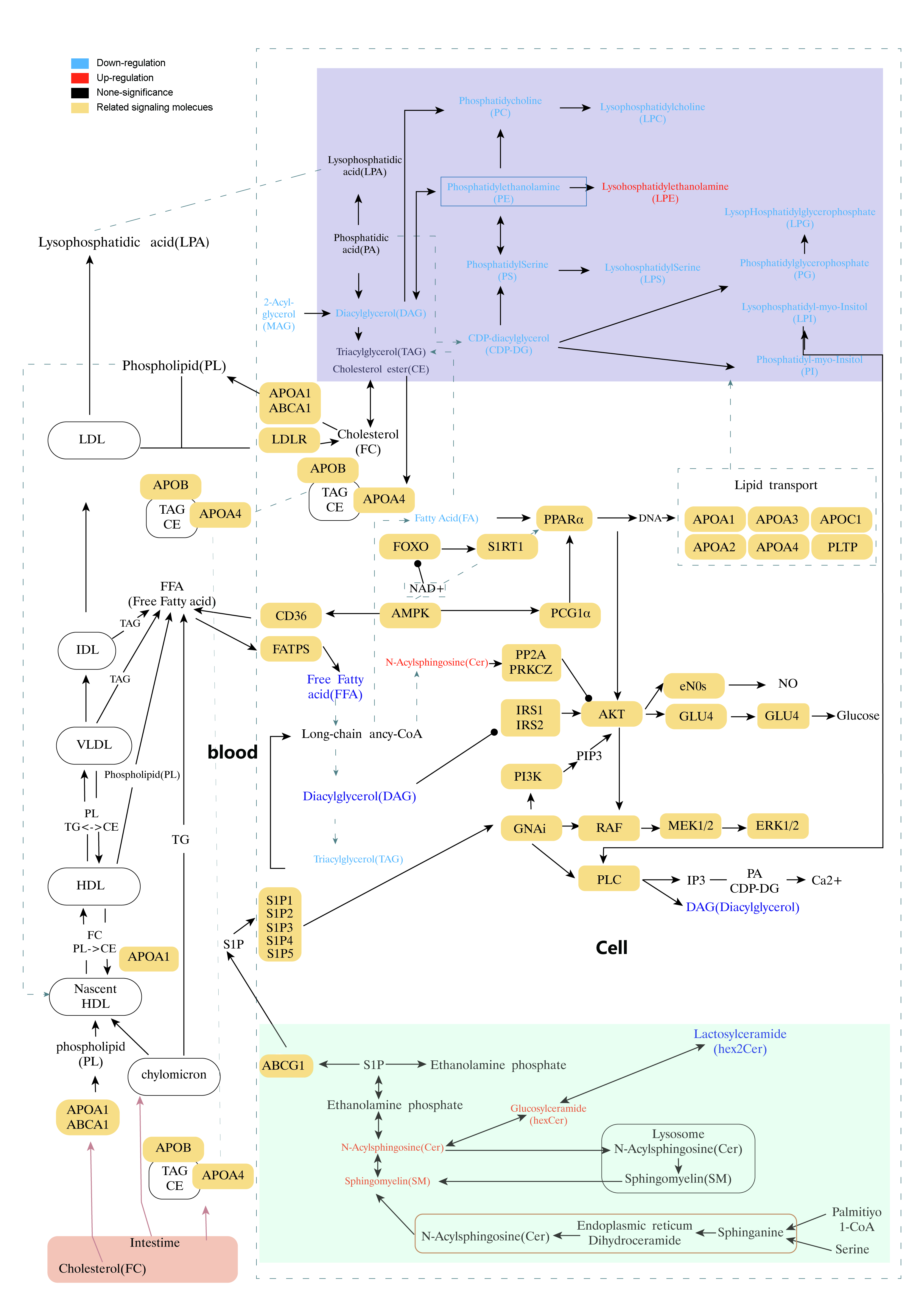


**Supplementary Figure 5.** **Molecular network mediated by resveratrol.** Resveratrol-mediated metabolic changes are revealed in three biological pathways including **(1)** the glycerolphospholipid metabolism pathway (upper panel), showing PC, PE, PS and CDP-DAG are down-regulated, possibly resulting in reduced PI and PG synthesis. (These glycerolphospholipids play important roles in transmembrane transport of substances between blood and peripheral cells). **(2)** Glycerolipid related insulin-resistance (IRS) pathway (middle panel), showing that reduced FA might stimulate AMPK which could result in PPARa activation and influence the regulation of lipid transport genes. Resveratrol mediated down-regulation of DAG might directly activate IRS/PI3K-AKT pathways. **(3)** Sphingolipid metabolism pathway (lower panel), showing that the synthesis of the sphingolipidsCer and SM are significantly increased. This may affect cell proliferation and apoptosis. The words in a blue font represent down-regulated lipids, in red, up-regulated lipids and black, lipids without significant changes. Yellow shading represents related signaling molecules.
