# Supplementary material for: Lipidomic Alterations and PPARα Activation Induced by Resveratrol Lead to Reduction in Lesion Size in Endometriosis Models
Source: Oxid Med Cell Longev. 2021 Sep 11;2021:9979953. doi: 10.1155/2021/9979953 (PMC8452402; doi:10.1155/2021/9979953)
Supplement: Supplementary Materials — Supplementary Figure 1: behavioral evaluation of model rats. Supplementary Figure 2: results of multivariate statistical analysis. Supplementary Figure 3: molecular characterization of related pathways. Supplementary Figure 4: molecular network mediated by resveratrol. Supplementary Table 1: a list of primers. [file 9979953.f1.zip › 9979953.f1/9979953.f2.docx]

| Supplementary Table 1. A list of primers | |  |
| --- | --- | --- |
| Gene | Primer sequence 5’-3’ | Primer sequence 3’-5’ |
| β-actin | ATCCGTAAAGACCTCTATGC | ACACAGAGTACTTGCGCTCA |
| PPARα | GGCAATGCACTGAACATCGAG | GAAAGCCGCTTGATAAGCCG |
| MMP2 | GTGACGGCTTCCTCTGGTGTTC | CAGGGCTGTCCATCTCCATTGC |
| VEGF | CGGTGTGGTCTTTCGTCCTTCTTAG | AGGGATGGGTTTGTCGTGTTTCTG |
| BCL-2 | TGGAGAGCGTCAACAGGGAGATG | GTGCAGATGCCGGTTCAGGTAC |
| ICAM-1 | CCTGGTCCTCCAATGGCTTCAAC | TCTGTGGGATGGATGGATACCTGAG |
| PCYT1a | CAGGGAGCGACGATGTGTATAAGC | ATGATGTCTGATGTGGAGATGCCTTC |
| CHPT1 | AGAGGCAGAAGCAGAGGCAGAG | TCCTGGCAGTCCTGGAACTTACTC |
| CEPT1 | TGGGCATCGGTCAACAAGGAAAC | AGTGGTGGTGTCGGTAACTGAAAC |
| EPT1 | GTCGTGGCAACTGTGATCGTAGG | CAGAGTGAAGGCAGCAGTGAGTGG |
| SPT | ACTGGAGGGACATCGTCAGCTAC | GCCAATCCGCAGCACCTTATCC |
| SGMS1 | GACTGGCTGCTGGAGAATGCTA | GAGGAGACTCGGCACAGAGGA |
| SMPD2 | GTTCTATTGTGTGGAGACCTCA | TAATCAATCCGGATACCAGACG |
| SPHK1 | GGGCTGCTCTGCTCTTTCTCAC | GGCTGGAACTTCTCACACTCTG |
| CERS2 | ACGCAGTGACAGAGAAGAAAC | GGCAGCTAGGACAACGGTTCA |
| DEGS1 | GCCTTCAGACAGCCAGAACTCA | GGACGCCTCAGTAACAGTCATA |
| ACER1 | GCTCTGCGAAAGTCTCTGCTCAC | CAAGTGTGCCCTGCTCTGTGTC |
| HMGCR | GACCAACCTTCTACCTCAGCAA | GGACAACTCACCAGCCATCACAG |
| NPC1L1 | ACAGGAACAAGAACAAGGCAG | AGGACAGTGCTAAGACGGTGA |
| NR1H3 | GTGCCTGATGTTTCTCCTGACTCTG | AAGTGTTGCCTCCCTGGTCTCC |
| CYP7A1 | TGGAAGAAGCGAACACTGGATGATG | CAGGAATGTGGGCAGCGAGAAC |
| LDLR | CTTGTCCATCTTCCTCCCCATTGC | ATCTCGTCCTCCGTGGTCTTCTG |
| GAPDH | CATGGCCTTCCGTGTTCCTA | CCTGCTTCACCACCTTCTTGAT |
